# Identification of Transglutaminase Reactive Residues in Human Osteopontin and Their Role in Polymerization

**DOI:** 10.1371/journal.pone.0113650

**Published:** 2014-11-24

**Authors:** Brian Christensen, Elias D. Zachariae, Carsten Scavenius, Morten Thybo, Morten M. Callesen, Søren Kløverpris, Claus Oxvig, Jan J. Enghild, Esben S. Sørensen

**Affiliations:** 1 Department of Molecular Biology and Genetics, Science Park, Aarhus University, Aarhus, Denmark; 2 Interdisciplinary Nanoscience Center, Aarhus University, Aarhus, Denmark; University of Texas MD Anderson Cancer Center, United States of America

## Abstract

Osteopontin (OPN) is a highly posttranslationally modified protein present in several tissues where it is implicated in numerous physiological processes. OPN primarily exerts its functions through interaction with integrins via the Arg-Gly-Asp and Ser-Val-Val-Tyr-Gly-Leu-Arg sequences located in the N-terminal part of the protein. OPN can be polymerized by the cross-linking enzyme transglutaminase 2 (TG2), and polymerization has been shown to enhance the biological activity of OPN. However, little is known about the reactivity and location of the glutamine and lysine residues involved in the TG2-mediated modification of OPN. Here we show that TG2 catalyses the incorporation of 5-(Biotinamido)pentylamine at glutamines in both the N- and C-terminal parts of OPN, whereas TG2 primarily incorporated the glutamine-donor peptide biotinyl-TVQQEL-OH into the C-terminal part of OPN. By mass spectrometric analyses we identified Gln34, Gln42, Gln193 and Gln248 as the major TG2 reactive glutamines in OPN. The distribution of reactive Gln and Lys residues in OPN proved to be important, as the full-length protein but not the physiologically highly active integrin-binding N-terminal part of OPN were able to polymerize in a TG2-mediated reaction. Collectively, these data provide important new molecular knowledge about the mechanism of OPN polymerization.

## Introduction

Osteopontin (OPN) is a multifunctional acidic phosphorylated glycoprotein containing an integrin binding Arg-Gly-Asp (RGD) sequence. OPN is a member of the SIBLING (Small Integrin-Binding LIgand N-linked Glycoprotein) protein family [1], [2] and is present in many different body fluids such as milk, urine and blood [3], [4] and it is also present as part of the extracellular matrix in *e.g.* bone [5]. OPN plays critical roles in physiological processes such as inflammation, tumorigenesis, mineralization and tissue remodeling [6]. Many of these versatile functions are exerted through interactions with integrin receptors via the RGD motif and a cryptic Ser-Val-Val-Tyr-Gly-Leu-Arg (SVVYGLR) sequence exposed upon proteolytic cleavage [6]. OPN undergoes many types of post-translational modification such as phosphorylation, glycosylation, proteolytic processing and transglutamination by transglutaminase 2 (TG2) or Factor XIIIa [4], [5], [7], [8]. Phosphorylation influences the ability of OPN to mediate RGD-dependent cell adhesion, stimulate bone resorption and inhibit mineralization [9], [10]. The significance of OPN glycosylation has not been well described, though a recent study suggests that *O*-glycosylation of OPN actually affects the phosphorylation of the protein and hence its interaction with cells [11]. Several matrix metalloproteases as well as plasmin and thrombin cleave OPN close to the RGD-sequence and thereby generates N-terminal fragments that show greater capability to mediate RGD-dependent cell attachment than the full-length protein [7], [12]. The presence of N-terminal OPN fragments has been shown during blood coagulation and in milk [7], [13] and the thrombin cleaved OPN is involved in rheumatoid arthritis [14], formation of renal calcium crystals [15] and stem cell retention in the bone marrow niche [16].

Polymerization of OPN by TG2 is another modification that alters the function of the protein. TG2, also known as tissue type transglutaminase, is a Ca^2+^-dependent protein-cross-linking enzyme that catalyzes the formation of a covalent, γ-glutamyl-ε-lysyl (isopeptide) bond between specific Lys and Gln residues of its substrate proteins [17], [18]. TG2 is ubiquitously expressed in mammalian tissues and is implicated in stabilization of extracellular matrix, cell adhesion and wound healing processes [17]. TG2 polymerized OPN has been identified *in vivo* in bone and calcified aorta [5], [19]. In bone, OPN forms homo-polymeric protein aggregates rather than polymerizing in a heterotypic manner with other proteins [5]. Such polymerization of OPN increases its collagen binding properties and enhances opsonization facilitated by the protein [20], [21]. *In vitro* studies have shown increased cell-binding properties of TG2-polymerized OPN compared to monomeric OPN leading to enhanced cell adhesion and migration [22], [23]. TG2-catalysed polymerization of OPN can form *de novo* binding sites for integrins, as the α_9_β_1_ integrin binds polymeric OPN independently of the SVVYGLR motif [24]. Recently, it has been shown that polymeric OPN is responsible for chemotactic recruitment of neutrophils *in vivo* [25]. These studies indicate that polymerization of OPN is an important regulatory mechanism in OPN's interaction with cells.

OPN contains several highly conserved Gln and Lys residues in both its N- and C-terminal parts. The conservation of these residues imply that they could be functionally important, as is the case for other highly conserved elements like the integrin-binding sequences, the poly-aspartic region and the sites of phosphorylation, glycosylation and proteolytic cleavage [9]. Hitherto only Gln34 and Gln36 in bovine OPN (conserved throughout mammalian species) have been identified as TG2 reactive [26]. However, OPN-c, a splice variant which does not contain Gln34 and Gln36, can still undergo TG2-mediated polymerization [25] indicating that there are more reactive Gln residues in OPN. Furthermore, the TG2 reactive Lys residues in OPN have not previously been studied. Thus, only little is known about the reactivity of the Gln and Lys residues involved in the TG2-mediated modification of OPN.

In this study, we identify the TG2 reactive Gln and Lys residues in both the N- and C-terminal parts of human OPN. The study provides novel structural information about the TG2-mediated polymerization of OPN, and indicates that the N-terminal part of OPN containing both the RGD- and SVYYGLR integrin binding motifs and which has been shown to be important in many physiological processes cannot be polymerized by TG2.

## Materials and Methods

### Materials

Sequencing grade modified trypsin was from Promega. Guinea pig tissue transglutaminase (TG2), bovine alkaline phosphatase, α-chymotrypsin and human thrombin were from Sigma. Biotinyl-TVQQEL-OH and 5-(Biotinamido)pentylamine were from Zedira. Monomeric avidin and MaxiSorp immunoassay plates were from Thermo Scientific. Vydac C_18_ reverse-phase resin was obtained from The Separations Group. The µRPC (narrow-bore reverse-phase chromatography) C2/C18 PC 2.1/10 and the Superdex 75 PC 3.2/30 columns were from GE Healthcare.

### Purification and thrombin cleavage of OPN

OPN was purified from human milk as described [27]. The purity of the protein was determined by N-terminal sequencing and SDS-PAGE. N- and C-terminal fragments of OPN were generated by digestion with thrombin (30 mU/µg OPN) in 0.1 M ammonium bicarbonate at 37°C for 1 h. The fragments were separated by reversed-phase high-performance liquid chromatography (RP-HPLC) on a Vydac C_18_ column connected to a GE Healthcare LKB system. Separation was carried out in 0.1% trifluoroacetic acid (buffer A) and eluted with a gradient of 75% 2-propanol in 0.1% trifluoroacetic acid (buffer B) developed over 80 min (0–5 min, 0% buffer B; 5–70 min, 0–50% buffer B; 70–80 min, 50–95% buffer B) at a flow rate of 0.85 ml/min. The fragments were detected in the effluent by measuring the absorbance at 226 nm.

### TG2-catalyzed incorporation of 5-(Biotinamido)pentylamine and biotinyl-TVQQEL-OH

MaxiSorp plates were coated with full-length OPN, the N-terminal part of OPN (Ile^1^-Arg^152^) or the C-terminal part of OPN (Ser^153^-Asn^298^) (3 µg/ml) in phosphate buffered saline (PBS) overnight at 4°C. Subsequently, the wells were blocked with 2% Tris-Tween buffer (2% Tween 20, 10 mM of Tris-HCl, 1 M of NaCl, 10 mM of CaCl_2_, pH 7.4) for 1 h at room temperature. A reaction mixture (0.1 ml) containing 10 µM biotinyl-TVQQEL-OH or 5-(Biotinamido)pentylamine, and 0, 0.25, 0.5, 1, 2, 3, 5 µg/ml TG2 in 40 mM of Tris-HCl (pH 8.3), 140 mM NaCl, 10 mM CaCl_2_, 5 mM dithioerythritol was added to the plates and incubated for 3 h at 37°C. Biotinyl-TVQQEL-OH and 5-(Biotinamido)pentylamine cross-linked to OPN were detected by incubation with horseradish peroxidase-conjugated streptavidin (Dako) (diluted 1∶8000) for 1 h at 37°C. Color development was obtained with TMB-one substrate (Kem-En-Tec) and the reaction was stopped by addition of 0.2 M H_2_SO_4_. Color intensity was measured at 450 nm using an ELISA reader.

### Identification of TG2 reactive residues

Recombinant human OPN or OPN purified from human milk were incubated with or without alkaline phosphatase (30 milliunits/µg OPN) in 10 mM ammonium bicarbonate (pH 8.5) overnight at 37°C. The samples were dialysed against 40 mM of Tris-HCl (pH 8.3), 140 mM NaCl, 10 mM CaCl_2_ followed by addition of dithioerythritol (to 5 mM) and 5-(Biotinamido)pentylamine or biotinyl-TVQQEL-OH (to 10 mM). Subsequently, the samples were incubated with TG2 (10∶1 w/w) at 37°C. After 15 min and 5 h, samples were taken and the reaction was stopped by addition of EDTA to a final concentration of 50 mM. The samples were dialyzed against 20 mM ammonium bicarbonate and digested with trypsin (1∶30 w/w) or chymotrypsin (1∶30 w/w, only for the biotinyl-TVQQEL-OH sample) for 5 h at 37°C followed by inactivation of the proteases by addition of phenylmethanesulfonyl fluoride (to 1.5 mM). Subsequently, the native human milk OPN was incubated with alkaline phosphatase as described above. The tryptic and chymotryptic peptides were lyophilized and dissolved in PBS and applied to a monomeric avidin affinity column equilibrated in 100 mM NaH_2_PO_4_ and 150 mM NaCl (pH 7.4). After extensive washing, the biotin-labeled peptides were eluted using 100 mM glycine (pH 2.8). The purified tryptic peptides labelled with 5-(Biotinamido)pentylamine were desalted on C_18_ stage tips (Proxion) before they were subjected to NanoESI-MS/MS analyses as described below. The purified tryptic and chymotryptic peptides labelled with biotinyl-TVQQEL-OH were desalted and concentrated on a Zip-tip column containing C_18_ reversed-phase material (Millipore) and analysed by MALDI-MS as described below.

For further analysis, monomeric avidin purified tryptic peptides of dephosphorylated milk OPN that had been incubated with TG2 and 5-(Biotinamido)pentylamine for 5 h were fractionated by reverse-phase HPLC on a µRPC C_2_/C_18_ PC 2.1/10 column connected to a GE Healthcare SMART system. Separation was carried out in 0.1% trifluoroacetic acid (buffer A) and eluted with a gradient of 60% acetonitrile in 0.1% trifluoroacetic acid (buffer B) developed over 54 min (0–9 min, 0% buffer B; 9–49 min, 0–50% buffer B; 49–54 min, 50–100% buffer B) at a flow rate of 0.15 ml/min. The peptides were detected in the effluent by measuring the absorbance at 214 nm. Peptides were characterized by MALDI- MS or MS/MS.

### Mass spectrometry

NanoESI-MS/MS analyses were performed on an EASY-nLC II system (ThermoScientific) connected to a TripleTOF 5600 mass spectrometer (AB Sciex) equipped with a NanoSpray III source (AB Sciex) operated under the control of Analyst TF version 1.5.1. The 5-(Biotinamido)pentylamine-labeled tryptic peptides were dissolved in 0.1% formic acid, injected, trapped and desalted isocratically on a Biosphere C18 column (5 µm, 2 cm×100 µm i.d.; Nano Separations). The peptides were eluted from the trap column and separated on a 15-cm analytical column (75 µm i.d.) packed in-house in a pulled emitter with RP ReproSil-Pur C18-AQ 3 µm resin (Dr. Marisch GmbH) a rate of 250 nL/min using a 30 min gradient from 5 to 35% phase B (90% acetonitrile in 0.1% formic acid).

The collected MS files were converted to Mascot generic format (MGF) using the AB SCIEX MS Data Converter beta 1.1 (AB SCIEX) and the “proteinpilot MGF” parameters. The generated peak lists were searched against the swiss-prot database using in-house Mascot search engine (matrix science). The following search parameters were used: *Homo sapiens*, trypsin, two missed cleavages, oxidation (Met) variable modification, and 2+, 3+, and 4+ charges. Peptide and MS/MS tolerances were set to 10 ppm and 0.2 Da, respectively. For reactive residue identification, pentylamine-biotin (Gln) variable modification was used. Three signature ion fragments at m/z 329, 395 and 440 arise from fragmentation at the site of biotin modification. All spectra were inspected manually. Peptides were only accepted if they contained three sequential y- or b-ions of high intensity and pentylamine-biotin signature ion fragments.

MALDI-MS analysis was performed using a Q-TOF Ultima MALDI mass spectrometer (Micromass/Waters Corporation) calibrated over the m/z range of 50–3000 using a polyethylene glycol mixture. External calibration of each MS spectrum was performed using Glu-fibrinopeptide B (m/z 1570.6774). The MS or MS/MS data obtained were processed using the Masslynx 4.0 software (Micromass). The theoretical peptide masses were calculated using the GPMAW program (Lighthouse Data).

### Multiple sequence alignment

Sequence analyses were performed on OPN from the following 25 mammalian species found in the UniProt database (UniProt release 2014_06): rat, *Rattus norvegicus* (P08721); mouse, *Mus musculus* (P10923); human, *homo sapiens* (P10451); bovine, *Bos tausrus* (P31096); pig, *Sus scrofa* (P14287); Rhesus monkey, *Macaca mulatta*, (F6V2X3); Chimpanzee, *Pan troglodytes* (H2RCV1); Lowland gorilla, *Gorilla gorilla gorilla* (G3QS39); Rabbit, *Oryctolagus cuniculus* (P31097); Sheep, *Ovis aries* (Q9XSY9); horse, *Equus caballus* (F7AYC1); cat, *Felis catus* (M3VZ83); dog, *Canis familiaris* (E2R161); african elephant, *Loxodonta africana* (G3SYB7); golden hamster, *Mesocricetus auratus* (Q0WX06); goat, *Capra hircus* (U5Y6U2); Little brown bat, *Myotis lucifugus* (G1PFK5); David's myotis, *Myotis davidii* (L5M309); Black flying fox, *Pteropus alecto* (L5KPG6); Brandt's bat, *Myotis brandtii* (S7MMZ5); Tasmanian devil, *Sarcophilus harrisii* (G3VEM2); White-tufted-ear marmoset, *Callithrix jacchus* (F7DN59); Thirteen-lined ground squirrel, *Spermophilus tridecemlineatus* (I3MYE1); Duckbill platypus, *Ornithorhynchus anatinus* (F7EC46); Gray short-tailed opossum, *Monodelphis domestica* (F7ANS8). The multiple protein sequence alignment was made using the alignment tool on the UniProt website.

### Cloning, expression, and purification of OPN variants

A construct containing residues 1–298 of human OPN (without the signal peptide) and a His-tag at the N-terminus [28] was amplified by polymerase chain reaction (PCR) using the 5′-primer cagaggatcccatcaccatcacc and the 3′-primer cgacctcgagctactaattgacc, and then cloned into the BamHI and XhoI sites within the multiple cloning site of the pGEX6p2 vector. An N-terminal fragment corresponding to plasmin cleavage of OPN (residues 1–154) was amplified from the previous full-length construct using the 5′-primer as above and the 3′-primer gaggaattcttattattttgacctcagtccataaacc, and then cloned into the BamHI and EcoRI sites within the multiple cloning site of the pGEX6p2 vector.

The plasmids were propagated in *E. coli* DH5α cells, and the constructs were verified by sequence analysis. Cells of *E. coli* strain BL-21cc were transformed with the expression vectors containing full-length OPN and the N-terminal part of OPN, respectively, using heat shock and grown on ampicillin-containing plates. Individual colonies were picked and propagated overnight in 50 ml of TY medium with 100 µg/ml ampicillin at 37 °C. 960 ml of TY medium with 100 µg/ml ampicillin and 2% glucose was inoculated with 40 ml of the bacteria, and incubated at 37 °C until OD600 reached 0.6, at which time isopropyl-1-thio-β-d-galactopyranoside was added to a final concentration of 0.1 mM. Cultures were grown for several more hours until OD600 reached 1.2–1.5, cells were collected and sonicated, and glutathione *S*-transferase fusion proteins were affinity-purified with glutathione-Sepharose 4B beads and then cleaved off from glutathione *S*-transferase with PreScission protease (Fisher Scientific). Purity of the product was confirmed by 18% SDS-polyacrylamide gel electrophoresis followed by Coomassie Blue staining. The recombinant OPN was further purified using a 1 ml metal-chelate affinity column (Qiagen) charged with nickel ions. Bound protein was eluted with 250 mM imidazole in PBS. The fractions containing OPN was identified by Western blotting and desalted on a PD10 column in 25 mM ammonium bicarbonate and lyophilized.

### Cross-linking reactions

Recombinant full-length OPN and the recombinant N-terminal part of OPN (250 µg/ml) were incubated with TG2 (25 µg/ml) in 40 mM of Tris-HCl (pH 8.3), 140 mM NaCl, 10 mM CaCl_2_ and 5 mM dithioerythritol at 37 °C. Reaction samples were quenched after 1 h, 3 h and 16 h by the addition of EDTA to 50 mM. The degree of TG2-mediated cross-linking was assessed by 18% SDS-PAGE followed by Coomassie Brilliant blue staining. The SDS-PAGE analysis was repeated four times.

In another experiment, recombinant full-length OPN and human milk OPN were incubated with TG2 in time series as above and the reactions were stopped with EDTA. The degree of cross-linking was assessed by 10% SDS-PAGE followed by Western blotting using a rabbit polyclonal anti-OPN antibody as described in [4].

## Results

### Human OPN contains TG2 reactive Gln and Lys residues

**Figure 1:**
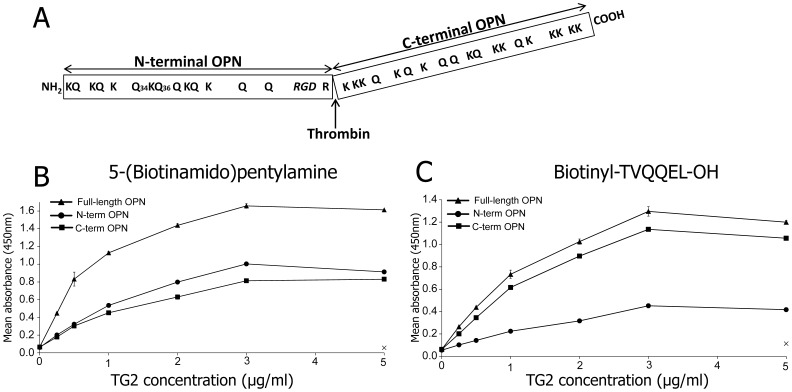
OPN contains TG2 reactive Gln and Lys residues. (A) Schematic representation of OPN showing the N- and C-terminal parts of the protein, the thrombin cleavage site and the distribution of potential TG2 reactive Gln and Lys residues. The previously identified TG2 reactive Gln residues (Gln34, Gln36) and the integrin binding RGD sequence are indicated. (B–C) Maxisorp plates were coated with full-length OPN (triangle), the N-terminal part of OPN (circle) or the C-terminal part of OPN (square) (3 µg/ml) and subsequently incubated with increasing concentrations of TG2 in the presence of the amine donor 5-(Biotinamido)pentylamine (B) or the amine acceptor biotinyl-TVQQEL-OH (C). As negative control, wells not coated with OPN but incubated with 5-(Biotinamido)pentylamine or biotinyl-TVQQEL-OH and TG2 (5 µg/ml) are indicated with a cross. The samples were incubated for 3 h at 37°C. For all experiments data are expressed as mean ±S.D. (n = 3). The experiments were repeated four times.

Gln34 and Gln36 in the N-terminal part of bovine OPN were the only TG2 reactive residues that have been unambiguously identified in any OPN sequence before this study (Figure 1A) [26]. To identify the TG2 reactive residues in human OPN and to test whether OPN also contains reactive Gln residues in the C-terminal part of the protein, OPN was cleaved with thrombin and the resulting N- and C-terminal fragments (Figure 1A) were separated by RP-HPLC as described [7]. Full-length as well as the N- and C-terminal parts of OPN were coated on a microtiter plate and co-incubated with 5-(Biotinamido)pentylamine and increasing concentrations of TG2. It was shown that 5-(Biotinamido)pentylamine was incorporated in both the N- and C-terminal parts of OPN in a concentration dependent manner (Figure 1B), indicating the presence of reactive Gln residues in both parts of the protein. The level of labeling was comparable between the N- and C-terminal parts of OPN.

To test whether OPN also contains TG2 reactive Lys residues, the different fragments were incubated with TG2 and the glutamine-donor peptide biotinyl-TVQQEL-OH (Figure 1C). The TG2-mediated incorporation of biotinyl-TVQQEL-OH demonstrated that OPN contains reactive Lys residues in both the N- and C-terminal parts of the protein, though the level of labeling of the N-terminal part was much lower than that of the C-terminal end.

### Identification of TG2 reactive Gln residues in OPN

OPN was incubated with TG2 in the presence of 5-(Biotinamido)pentylamine for five hours followed by digestion with trypsin and purification of the labeled peptides using a monomeric avidin resin. The purified peptides were then subjected to highly sensitive nLC-MS/MS analyses and the resulting mass spectra were searched against the Swiss-Prot database using a local Mascot search engine. Using this approach, we identified 12 Gln residues in OPN to be reactive in the transamidation reaction catalyzed by TG2 (Table 1). Of all the Gln residues in OPN only Gln84 and Gln103, which are located in a large acidic fragment not cleavable by trypsin, were not observed in this study. The peptides identified as containing reactive Gln residues are listed in Table 1.

**Table 1:** Identification of reactive Gln residues in OPN.

	Reactive Gln	Peptide	ppm	Spectral counts	Conservation
15 min	42	QNLLAP**Q**NAVSSEETNDFK	3.7	4	25
	193	AIPVA**Q**DLNAPSDWDSR	6.5	4	16
	248	KANDESNEHSDVIDS**Q**ELSK	4.3	1	23
5 h	5	**Q**ADSGSSEEK	3.6	5	11
	15	**Q**LYNK	0.3	1	18
	34	YPDAVATWLNPDPS**Q**K	7.1	12	24
	36	**Q**NLLAPQNAVSSEETNDFK	5.3	2	25
	42	QNLLAP**Q**NAVSSEETNDFK	3.3	8	25
	36/42	**Q**NLLAP**Q**NAVSSEETNDFK	3.6	1	25/25
	55	**Q**ETLPSK	5.5	5	25
	164	RPDI**Q**YPDATDEDITSHMESEELNGAYK	3.0	2	22
	193	AIPVA**Q**DLNAPSDWDSR	8.4	9	16
	213	GKDSYETS**Q**LDDQSAETHSHK	5.2	7	23
	217	GKDSYETSQLDD**Q**SAETHSHK	4.9	5	4
	226	GKDSYETSQLDDQSAETHSHK**Q**SR	3.6	1	12
	248	KANDESNEHSDVIDS**Q**ELSK	5.3	11	23

OPN was labeled with 5-(Biotinamido)pentylamine for 15 min or 5 h by TG2. Identified reactive Gln residues are underlined and shown in bold. The ppm differences between the measured and expected masses are listed. The spectral counts include all identified peptides containing the specific reactive residue. Conservation shows how many of the 25 mammalian OPN sequences used in a multiple sequence alignment that contains the specific reactive Gln residue. 5-(Biotinamido)pentylamine labeling and MS analyses were repeated four times.

Spectral counts (listed in Table 1) depend on both the ionization efficiency and the concentration of the analyzed peptides. It is therefore not an exact quantitative measure of peptide abundance but it can provide a rough indication of the peptide abundance and consequently the residue reactivity. None of the labeled Gln residues stood out in the spectral count numbers clearly marking them as major TG2 reactive residues after 5 h incubation with TG2, however, Gln34, Gln42, Gln193 and Gln248 were observed with the highest numbers. In order to get more information about which of the identified Gln residues that could be considered as the major TG2 reactive residues, we used two different approaches. Initially, OPN was incubated with 5-(Biotinamido)pentylamine and TG2 for only 15 min followed by nLC-MS/MS analysis as described above. After this short incubation time, only Gln42, Gln193 and Gln248 were labelled with the probe, indicating that these residues are major TG2 reactive sites in OPN (Table 1). In another experiment, OPN was labeled with 5-(Biotinamido)pentylamine for 5 h and the peptides were purified using the monomeric avidin resin and then further fractionated using reverse-phase HPLC. The fractions were analyzed by MALDI-MS or MS/MS resulting in identification of only four peptides containing 5-(Biotinamido)pentylamine corresponding to modification of Gln34, Gln42, Gln193 and Gln248 (Table 2). In summary, this shows that most Gln residues in OPN are able to incorporate the probe in a TG2-catalyzed reaction, but the major reactive residues and hence probably the biologically most relevant residues are Gln34, Gln42, Gln193 and Gln248.

**Table 2:** Identification of major reactive Gln residues in OPN by MALDI-MS and MS/MS.

Reactive Gln	Peptide	Measured	Expected	ppm
34	YPDAVATWLNPDPS**Q**K	2113.02	2113.04	11
42	QNLLAP**Q**NAVSSEETNDFK	2416.19	2416.17	−4
193	AIPVA**Q**DLNAPSDWDSR	2166.05	2166.06	7
248	KANDESNEHSDVIDS**Q**ELSK	2556.19	2556.18	−1

Monoisotopic molecular masses (MH^+^) were measured by MALDI-MS. The expected masses (MH^+^) include the 5-(Biotinamido)pentylamine modification and were calculated using the GPMAW software. The ppm differences between the measured and expected masses are listed. 5-(Biotinamido)pentylamine labeling, peptide separation and MS analyses were repeated four times.

To simplify the MS analysis, the identification of reactive Gln residues described above was performed on dephosphorylated human milk OPN. To test whether the reactivity of the Gln residues were influenced by phosphorylation of the protein, native human milk OPN was incubated for 15 min or 5 h with 5-(Biotinamido)pentylamine and TG2, before it was dephosphorylated and subsequently analyzed by nLC-MS/MS (Table S1). After 15 min, Gln34, Gln42 and Gln193 were identified as labeled with 5-(Biotinamido)pentylamine and after incubation for 5 h nine TG2-reactive Gln residues were identified including the four major reactive residues. An identical result was obtained with bacterial expressed recombinant OPN where ten TG2-reactive Gln residues were identified (Table S1).

A multiple sequence alignment of 25 mammalian OPN sequences found in the UniProt database (see Figure S1) showed that three out of the 12 identified TG2 reactive Gln residues; Gln36, Gln42 and Gln55 were fully conserved (Table 1). Furthermore, Gln34 was conserved in all species except for one and Gln213 and Gln248 were only missing in two of the 25 sequences. Thus, the major reactive Gln residues were also among the highly conserved residues in OPN.

### Identification of TG2 reactive Lys residues in OPN

**Figure 2:**
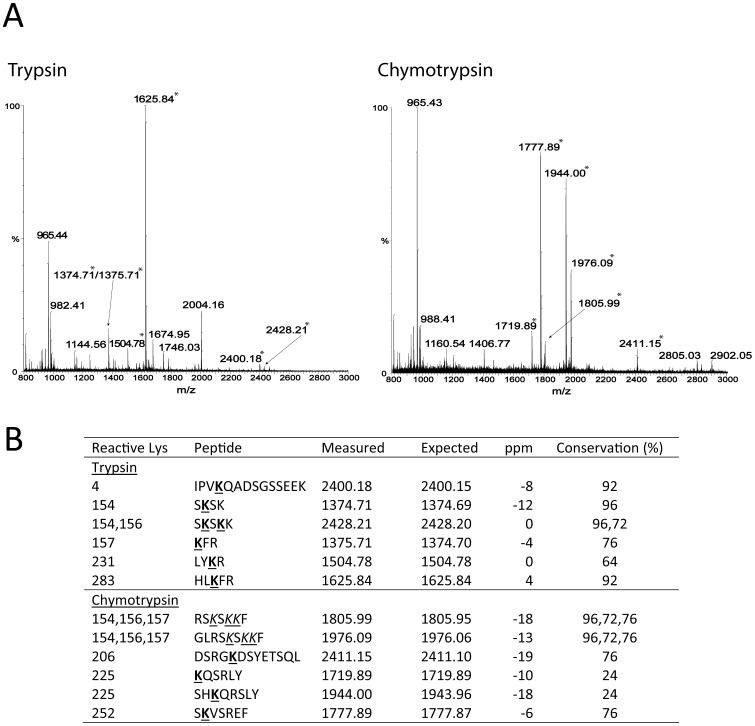
Identification of TG2 reactive lysines by mass spectrometry. (A) MALDI-MS of tryptic and chymotryptic peptides. Peptides observed with a mass corresponding to incorporation of biotin-TVQQEL are indicated with asterisks. (B) Table of tryptic and chymotryptic peptides containing biotin-TVQQEL. Location on the modified Lys residue in peptides containing more than one Lys was in some cases unambiguously achieved with the consideration that modified Lys residues are not trypsin substrates. The labeled Lys residues have been underlined and are shown in bold if the residue was unambiguously assigned. If the biotin label could not be assigned to a specific residue, the possible labeling sites are shown underlined and in italics. Monoisotopic molecular masses (MH+) were measured by MALDI-MS. The expected masses (MH+) include the biotinyl-TVQQEL-OH modification and were calculated using the GPMAW software. The ppm differences between the measured and expected masses are listed. Conservation shows how many of the 25 mammalian OPN sequences used in a multiple sequence alignment that contains the specific reactive Lys residue. Biotinyl-TVQQEL-OH labeling and MS analysis were repeated twice.

**Figure 3:**
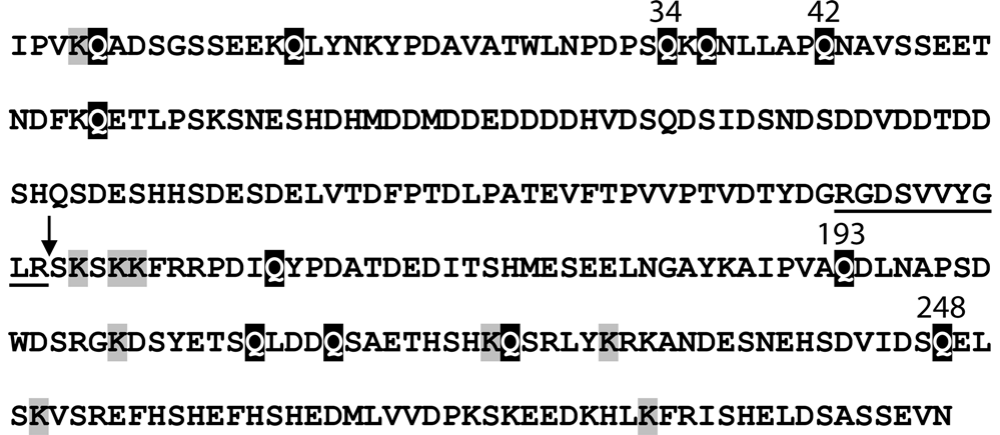
Identified TG2 reactive residues in OPN. TG2-reactive glutamines (black) and lysines (grey) are highlighted. The major reactive Gln residues are indicated with numbers. The thrombin cleavage site is indicated with an arrow and the integrin binding RGD-sequence and the cryptic integrin binding site SVVYGLR are underlined.

TG2 reactive Lys residues were identified by the same method as described for the reactive Gln residues using the Gln-donor peptide, biotinyl-TVQQEL-OH as probe. However, biotinylated OPN was subjected to proteolytic digestion with either trypsin or chymotrypsin before purification on the monomeric avidin resin and then analyzed by MALDI-MS without further purification (Figure 2). By MS analysis of tryptic and chymotryptic peptides Lys4, Lys154, Lys156, Lys157, Lys206, Lys225, Lys231, Lys252 and Lys283 were identified as TG2 reactive residues. The multiple sequence alignment (Figure S1) showed that Lys4, Lys154 and Lys283 were the best conserved among the identified reactive Lys residues. Lys154 was present in all species except one and Lys4 and Lys283 were only missing in two of the 25 sequences. The combined results of the MS and MS/MS analyses of the identified TG2 reactive Gln and Lys residues are shown in Figure 3.

### Polymerization of full-length OPN and of the N-terminal part of OPN

Although full-length OPN has been shown to be a TG2 substrate and to polymerize in a TG2-mediated reaction both *in vivo* and *in vitro*, the TG2-mediated cross-linking of the naturally occurring and highly active N-terminal fragment of OPN resulting from thrombin cleavage (Figure 1A) has never been investigated. The N-terminal part contains several highly conserved TG2 reactive Gln residues, whereas the majority of reactive Lys residues are located in the C-terminal part of the protein, which could influence its ability to be polymerized by TG2. To compare the effect of TG2 on full-length OPN and the N-terminal fragment, both were incubated with TG2 in a time series and the reaction products were analyzed by SDS-PAGE.

**Figure 4:**
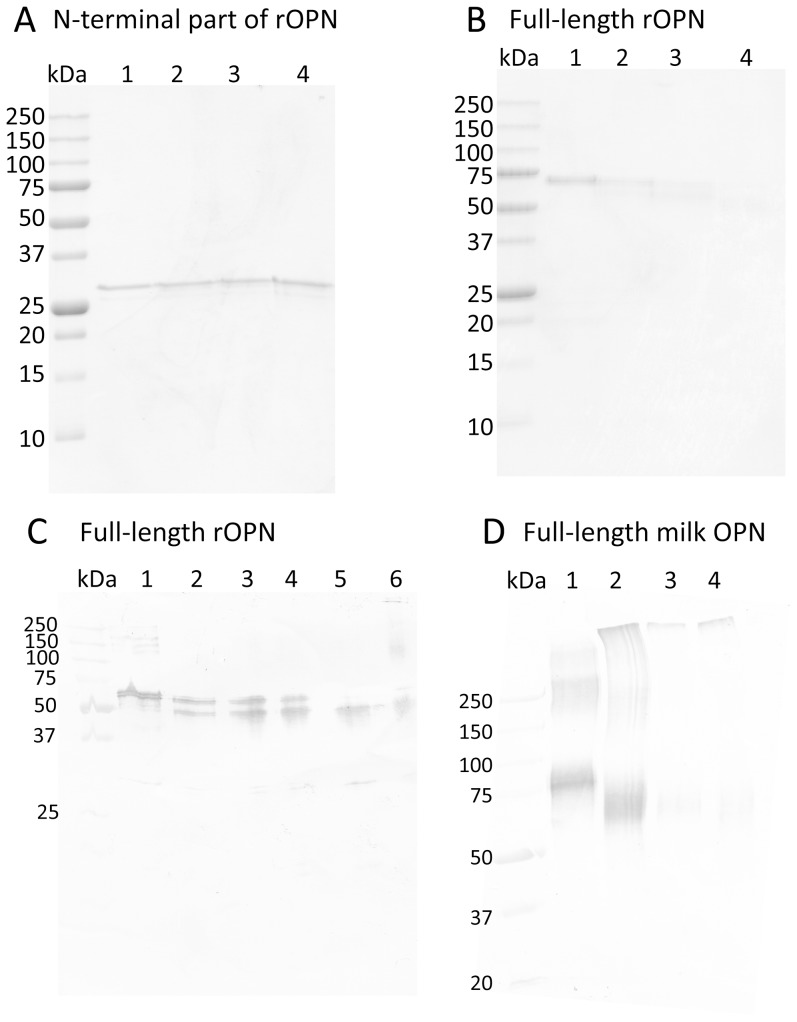
Polymerization of full-length OPN and the N-terminal part of OPN. The N-terminal part of recombinant OPN (A), full-length recombinant OPN (B–C) and human milk OPN (D) were incubated with TG2 in a 10∶1 ratio (w/w). In A and B, proteins were separated by 18% SDS-PAGE and stained with Coomassie Brilliant blue. In C and D, proteins were separated by 10% SDS-PAGE and detected by a polyclonal OPN antibody. A, B and D; OPN without TG2 (lane 1), or TG2-treated for 1 h (lane 2), 3 h (lane 3) and 16 h (lane 4). C, OPN without TG2 (lane 1), or TG2-treated for 15 min (lane 2), 30 min (lane 3), 1 h (lane 4), 3 h (lane 5) and 16 h (lane 6).

The 18% SDS-PAGE gels show that the N-terminal part of OPN migrated with the same mobility independently of the presence of TG2 or the incubation time indicating that this fragment cannot polymerize (Figure 4A). Full-length recombinant OPN migrated as expected as a single band before incubation with TG2, but after 1 h and 3 h of incubation with TG2, a second faster migrating band appeared probably representing intramolecularly cross-linked OPN as has been observed in other studies [22], [25]. After 16 h incubation, both bands almost disappeared presumably because of polymerization into high-molecular weight forms. We were not able to observe the polymerized form of full-length OPN by Coomassie Brilliant Blue staining in the 18% SDS-PAGE gels used (Figure 4B, lane 4). However, when analyzing a time series of TG2-treated full-length OPN by 10% SDS-PAGE followed by Western blotting, OPN polymer bands were observed migrating at a molecular weight corresponding to approximately 150 kDa and at the interface between the stacking and the resolving gel after 16 h incubation (Figure 4C, lane 6) indicating that OPN is polymerizing after TG2 treatment. To test whether phosphorylation affects the polymerization reaction, phosphorylated milk OPN was incubated with TG2. After 1 h, bands of both intramolecular cross-linked OPN and polymeric OPN were observed (Figure 4D, lane 2). After longer incubation times, the intra-molecular cross-linked OPN disappeared whereas the polymeric OPN band was still present (Figure 4D, lane 3 and 4).

## Discussion

In the present study, we have shown that human OPN contains TG2 reactive Gln residues in both the N- and C-terminal parts of the protein. Based on the incorporation of 5-(Biotinamido)pentylamine (Figure 1B), it is estimated that the two fragments contain a comparable number of reactive Gln residues. By using highly sensitive nLC-MS/MS, a total of 12 out of 14 Gln residues in OPN were shown to be acceptor sites for TG2-mediated incorporation of the probe. Using the same approach, 23 out of 90 Gln residues in human complement C3 have previously been identified as targets for TG2 [29]. This shows that though nLC-MS/MS is an extremely sensitive technique, not all Gln residues in substrate proteins are identified as targets for TG2 as is the case for OPN in the present study. So far, no consensus sequence for TG2 reactive Gln residues has been established, although there are reports showing that a proline residue in the vicinity of the reactive Gln residue is important for the recognition, especially in the motif Gln-xxx-Pro [30], [31]. Most of the identified reactive Gln residues in OPN are located in the near vicinity of a proline residue. It has also been suggested that the overall structure of the TG2 substrate proteins, rather than the primary structure around the Gln residue is important for the reactivity [30]. In that regard, it has been considered that TG2 preferably uses Gln residues that are located in disordered regions of proteins [32]. Biophysical studies have consistently demonstrated that OPN is an intrinsically disordered protein without any ordered structure [1], thereby all its Gln residues are potential TG2 reactive sites.

Based on the number of times the individual reactive residues have been identified and experiments using short TG2 reaction times (Table 1) and a less sensitive HPLC approach, we found the highest TG2 preference for Gln34, Gln42, Gln193 and Gln248. It is therefore plausible that these residues are the most relevant target sites *in vivo*. In a multiple protein sequence alignment of 25 mammalian OPN sequences in which Gln34, Gln42 and Gln248 were found to be highly conserved throughout the analyzed species (Table 1 and Figure S1). Several functional important motifs like phosphorylation sites, glycosylation sites, the RGD-sequence and the cryptic integrin binding motif SVVYGLR are highly conserved. The conservation of these Gln residues suggests that they may also be important for the function of OPN.

The reactivity of Gln residues was not affected by the phosphorylation or glycosylation status of OPN, as practically identical results were obtained in the nLC-MS/MS analysis of native phosphorylated and glycosylated human milk OPN, dephosphorylated human milk OPN and recombinant (non-modified) OPN. This is in agreement with the formation of both intra-molecular cross-linked monomers and polymers of both recombinant and milk OPN after TG2-treatment (Figure 4) and with other studies that show TG2-reactivity of both phosphorylated OPN from milk and bone, as well as bacterial expressed OPN [5], [20], [23], [25], [26].

Incorporation of the glutamine-donor peptide biotinyl-TVQQEL-OH showed that the C-terminal part of OPN contains the majority of the TG2 reactive Lys residues in OPN (Figure 1C). MALDI-MS of tryptic and chymotryptic peptides also indicated that the majority of reactive Lys residues were located in the C-terminal part, as Lys4, Lys154, Lys156, Lys157, Lys206, Lys225, Lys231, Lys252 and Lys283 were identified as TG2 reactive residues. Identification of reactive Lys residues is not as straightforward as the localization of reactive Gln residues. The biotinyl-TVQQEL-OH probe is a peptide which hampers ionization in MS and complicates MS/MS interpretations compared to incorporation of the simpler 5-(Biotinamido)pentylamine probe. Furthermore, biotinyl-TVQQEL-OH is attached to Lys residues that subsequently cannot be cleaved by trypsin which results in larger peptides for the MS analysis. As the C-terminal part contains more Lys residues and several Arg residues advantageous for trypsin cleavage compared to the N-terminal part, it is not surprising that the MS analysis of tryptic peptides resulted in localizing of more reactive Lys residues in the C-terminal part (Figure 2). However, the MS analysis of the chymotryptic peptides only resulted in identification of reactive Lys residues in the C-terminal part. In summary, these data show that the TG2 reactive Lys residues in OPN are predominately located in the C-terminal part of the protein, though it cannot be excluded from this study that the N-terminal part contains more reactive Lys residues than Lys4. TG2 is much less selective towards amine donor Lys residues than it is with regard to the Gln substrates, and no consensus sequence for TG2 reactive Lys residues has been deduced [30]. However, when comparing 30 different amine donors in characterized TG2 substrate proteins, a preference for Ser, Gln and Lys as the amino acid directly preceding the reactive Lys residue has been suggested to enhance reactivity [33]. Lys154, Lys156 and Lys157 are located in such sequences (Figure 3) and these residues were also present in five out of twelve peptides containing the biotinyl-TVQQEL-OH probe (Figure 2).

Polymerization of OPN by TG2 is physiologically relevant as it alters the functionality of the protein and especially its integrin binding properties [22]–[25]. Interestingly, OPN seems to form homo-polymer protein aggregates rather than polymerizing in a heterotypic manner with other proteins [5]. By site-directed mutagenesis it has been shown that especially Gln34 and/or Gln36 and to a lesser extent Gln42 and Gln55 play important roles in TG2-mediated homo-polymerization of OPN [25]. These Gln residues in the N-terminal part are among the best conserved residues in OPN, as all 25 aligned sequences contained Gln36, Gln42 and Gln55 whereas Gln34 is only missing in the African elephant (Figure S1). In the present study, Gln34 and Gln42 are among the most TG2 reactive residues in OPN which also suggests an important role of these residues for the TG2 regulated function of OPN. Our data suggests that Lys residues in the C-terminal part most likely are the amine donors in the polymerization, as the N-terminal fragment of OPN was unable to polymerize. This observation is interesting, as the N-terminal fragment of OPN contains the integrin recognition motifs and that specific cleavage by *e.g*. thrombin and plasmin modulates the function of OPN and can lead to enhanced cell adhesion, migration and resistance to apoptosis [7], [9], [34]. Cleaved OPN has been suggested to play a role in a variety of biological processes such as cancer development [34], rheumatoid arthritis [14], formation of renal calcium crystals [15] and stem cell retention in the bone marrow niche [16]. Thus, the altered ability of the N-terminal part of OPN to polymerize when compared to the full-length protein may be of importance *in vivo*.

In the present study we have identified the Gln residues in OPN that are substrates for TG2. The major reactive residues were shown to be Gln34, Gln42, Gln193 and Gln248. OPN also contained several TG2 reactive Lys residues that were predominately located in the C-terminal part of the protein. Polymerization of OPN seemed to involve isopeptide bonds between residues located in the N-terminal part (probably Gln residues) and the C-terminal part (probably Lys residues). This can have important biological consequences for the activity of the highly active N-terminal fragment as it is not able to polymerize *in vivo.*

## Supporting Information

Figure S1 **Alignment of mammalian OPN sequences.** Sequence analyses were performed on OPN from the following 25 mammalian species found in the UniProt database (UniProt release 2014_06). The TG2-reactive glutamine (black) and lysine (grey) residues identified in this study are highlighted and numbered. Human, homo sapiens (P10451); rat, Rattus norvegicus (P08721); mouse, Mus musculus (P10923); bovine, Bos tausrus (P31096); pig, Sus scrofa (P14287); Rhesus monkey, Macaca mulatta, (F6V2X3); Chimpanzee, Pan troglodytes (H2RCV1); Lowland gorilla, Gorilla gorilla gorilla (G3QS39); Rabbit, Oryctolagus cuniculus (P31097); Sheep, Ovis aries (Q9XSY9); horse, Equus caballus (F7AYC1); cat, Felis catus (M3VZ83); dog, Canis familiaris (E2R161); african elephant, Loxodonta africana (G3SYB7); golden hamster, Mesocricetus auratus (Q0WX06); goat, Capra hircus (U5Y6U2); Little brown bat, Myotis lucifugus (G1PFK5); David's myotis, Myotis davidii (L5M309); Black flying fox, Pteropus alecto (L5KPG6); Brandt's bat, Myotis brandtii (S7MMZ5); Tasmanian devil, Sarcophilus harrisii (G3VEM2); White-tufted-ear marmoset, Callithrix jacchus (F7DN59); Thirteen-lined ground squirrel, Spermophilus tridecemlineatus (I3MYE1); Duckbill platypus, Ornithorhynchus anatinus (F7EC46); Gray short-tailed opossum, Monodelphis domestica (F7ANS8).
(DOCX)

Table S1 **Identification of reactive Gln residues in human milk OPN and recombinant OPN.** OPN was labeled with 5-(Biotinamido)pentylamine for 15 min or 5 h by TG2. Identified reactive Gln residues are underlined and shown in bold. The ppm differences between the measured and expected masses are listed.
(DOCX)
