# Supplementary material for: Identification of Transglutaminase Reactive Residues in Human Osteopontin and Their Role in Polymerization
Source: PLoS One. 2014 Nov 24;9(11):e113650. doi: 10.1371/journal.pone.0113650 (PMC4242673; doi:10.1371/journal.pone.0113650)
Supplement: Figure S1 — Alignment of mammalian OPN sequences. Sequence analyses were performed on OPN from the following 25 mammalian species found in the UniProt database (UniProt release 2014_06). The TG2-reactive glutamine (black) and lysine (grey) residues identified in this study are highlighted and numbered. Human, homo sapiens (P10451); rat, Rattus norvegicus (P08721); mouse, Mus musculus (P10923); bovine, Bos tausrus (P31096); pig, Sus scrofa (P14287); Rhesus monkey, Macaca mulatta, (F6V2X3); Chimpanzee, Pan troglodytes (H2RCV1); Lowland gorilla, Gorilla gorilla gorilla (G3QS39); Rabbit, Oryctolagus cuniculus (P31097); Sheep, Ovis aries (Q9XSY9); horse, Equus caballus (F7AYC1); cat, Felis catus (M3VZ83); dog, Canis familiaris (E2R161); african elephant, Loxodonta africana (G3SYB7); golden hamster, Mesocricetus auratus (Q0WX06); goat, Capra hircus (U5Y6U2); Little brown bat, Myotis lucifugus (G1PFK5); David's myotis, Myotis davidii (L5M309); Black flying fox, Pteropus alecto (L5KPG6); Brandt's bat, Myotis brandtii (S7MMZ5); Tasmanian devil, Sarcophilus harrisii (G3VEM2); White-tufted-ear marmoset, Callithrix jacchus (F7DN59); Thirteen-lined ground squirrel, Spermophilus tridecemlineatus (I3MYE1); Duckbill platypus, Ornithorhynchus anatinus (F7EC46); Gray short-tailed opossum, Monodelphis domestica (F7ANS8). (DOCX) [file pone.0113650.s001.docx]

SP|P10451|OSTP_HUMAN -------------------MRIAVICFCLLGITCAIPVK----QADSGSSEEKQLYNKYP 21

15

5

4

-16

1

SP|P08721|OSTP_RAT -------------------MRLAVVCFCLFGLASCLPVK----VAEFGSSEEKAHYSKHS

SP|P10923|OSTP_MOUSE -------------------MRLAVICFCLFGIASSLPVK----VTDSGSSEEK-LYSLHP

SP|P31096|OSTP_BOVIN -------------------MRIAVICFCLLGIASALPVK----PTSSGSSEEKQLNNKYP

SP|P14287|OSTP_PIG -------------------MRIAVIAFCLWGFASALPVK----QTNSGSSEEKLLSNKYT

TR|F6V2X3|F6V2X3_MACMU -------------------MRIAVISFCLLGIAYALPVK----QADSGSSEEKQLYNKYP

TR|H2RCV1|H2RCV1_PANTR -------------------MRIAVICFCLLGITCALPVK----QADSGSSEEKQLYNKYP

TR|G3QS39|G3QS39_GORGO -------------------MRIAVICFCLLGITYALPVK----QADSGSSEEKQLYNKYP

SP|P31097|OSTP_RABIT -------------------MRIAVICFCLLGMAYALPVK----HADSGSSEEKQLYHKHP

SP|Q9XSY9|OSTP_SHEEP -------------------MRIAVICFCLLGIASALPVK----PTSSGSSEEKQLNNKYP

TR|F7AYC1|F7AYC1_HORSE -------------------MRIAVICLCLLGIAYALPVN----QADSGSSEEKQLYNKHS

TR|M3VZ83|M3VZ83_FELCA -------------------MRIAVICFCLLGIAYAIPIK----QTDSGSSEEKQLYNKYP

TR|E2R161|E2R161_CANFA -------------------MRIAVICFCLLGIAYAIPIK----HADSGSSEEKQLYNKYP

TR|G3SYB7|G3SYB7_LOXAF -------------------MRTAVICFCLLGIAYALPVKY---QADSGSSEEKQLHSKHP

TR|Q0WX06|Q0WX06_MESAU -------------------MRFAVICFCLFGIASSLPVK----VADSGSSEEKLHYSKHT

TR|U5Y6U2|U5Y6U2_CAPHI -------------------MRIAVICFCLLGIASALPVK----PTSSGSSEEKLLNNKYP

TR|G1PFK5|G1PFK5_MYOLU MGIVPRSLDKKTQGSIQTNRIKANIEF--PG------VT----VLDCLMKTLRELYNKHP

TR|L5M309|L5M309_MYODS ------------------MRI-AVICFCLLGITSALPVK----HADSGSSEEKQLYNKHP

TR|L5KPG6|L5KPG6_PTEAL -------------------MRIAVICFCLVGMAYALPVK----HADSGSSEERQLFNKYP

TR|S7MMZ5|S7MMZ5_MYOBR ------------------MRI-AVICFCLLGIAYALPVK----HADSGSSEERQLYNKHP

TR|G3VEM2|G3VEM2_SARHA ------------------MMRTAVICFCLLGIVSALPVKQ---QINSGSSEEKQLYSKHP

TR|F7DN59|F7DN59_CALJA -------------------MRIAVICFCLLGIAYALPVK----RADSGSSEEKQLYNKYP

TR|I3MYE1|I3MYE1_SPETR -------------------MRFAVIFFCLVGIAYSFPVK----QADSGSSEERQLYNKHP

TR|F7EC46|F7EC46_ORNAN -------------------MRTAVICLCLISIACALPVKRSGPEANSGSSEEKQLYNKHP

TR|F7ANS8|F7ANS8_MONDO -------------------MRTAVICFCLLGIVSALPVKH---QINSGSSEERRLYNKHP

55

42

34 36

SP|P10451|OSTP_HUMAN DAVATWLNPDPSQKQNLLAPQNAVSSEETN-DFKQETLPSKSNESHDHMDDMD--DEDDD 78

SP|P08721|OSTP_RAT DAVATWLKPDPSQKQNLLAPQNSVSSEETD-DFKQETLPSNSNESHDHMDDDDD-DDDDG

SP|P10923|OSTP_MOUSE DPIATWLVPDPSQKQNLLAPQNAVSSEEKD-DFKQETLPSNSNESHDHMDDDDDDDDDDG

SP|P31096|OSTP_BOVIN DAVATWLKPDPSQKQTFLAPQNSVSSEETD-DNKQNTLPSKSNESPEQTDDLD--DDDDN

SP|P14287|OSTP_PIG DAVATLLKPDPSQKQTFLAPQNTISSEETD-DFKQETLPSKSNESPEQTDDVD--DDDDE

TR|F6V2X3|F6V2X3_MACMU DAVATWLKPDPSQKQNLLAPQNAVSSEETN-DFKQETLPSKSNESHDHMDDVD--DEDDD

TR|H2RCV1|H2RCV1_PANTR DAVATWLNPDPSQKQNLLAPQNAVSSEETN-DFKQETLPSKSNESHDHMDDVD--DEDDD

TR|G3QS39|G3QS39_GORGO DAVATWLNPDPSQKQNLLAPQNAVSSEETN-DFKQETLPSKSNESHDHMDDVD--DEDDD

SP|P31097|OSTP_RABIT DALATWLNPDPSQKQNLLTPQNAMSSEEKD-DLKQETLPSKSIESHDHMDDID--EDEDD

SP|Q9XSY9|OSTP_SHEEP DAVATWLKPDPSQKQTFLEPQNSVSSEETD-DNKQNTLPSKSNESPEQTDDLD--DDDEN

TR|F7AYC1|F7AYC1_HORSE DAVSIWLKPDPSQKQNLLAPQ-TVSSEETD-NLKQETLPSQSNESHDHTDDVD--DDDVG

TR|M3VZ83|M3VZ83_FELCA VAVATWPKPDPSQKQTFLALQNAVLSEETD-DFKQKTLASKSNESHDV----D--DEDDE

TR|E2R161|E2R161_CANFA GAVATWLKPDPSQKQTFLALQNAVLTEETD-DFKQKTFSSKSNESHDD----V--DEDDG

TR|G3SYB7|G3SYB7_LOXAF DTVATWLKPDVSYKQILLAPQNAVSSEEND-KLKQETLPSKSNESHDQTDNVD--DEDDG

TR|Q0WX06|Q0WX06_MESAU DAVATWLEPDPSQKQNLLAPQNVVSSEETD-DLKQETLPSNSNESHDHMDDDND-DDDDG

TR|U5Y6U2|U5Y6U2_CAPHI DAVATWLKPDPSQKQTFLEPQNSVSSEETD-DNKQNTLPSKSNESPEQTDDLD--DDDET

TR|G1PFK5|G1PFK5_MYOLU DAVATWLKPDPSQKQSLLAPQNTVSSEETD-DFKQETLPSKSNESQDHTDDVG--DD-DE

TR|L5M309|L5M309_MYODS DAVATWLKPDPSQKQSLLAPQNTVSSEETD-DFKQETLPSQSNESQDHTDDVD--DDDDE

TR|L5KPG6|L5KPG6_PTEAL DAVATWLKPDPSQKQNLLAPQNTVSSEETD-NFKQETLPSQSNESHDHTDDVD--DEDDE

TR|S7MMZ5|S7MMZ5_MYOBR DAVATWLKPDPSQKQSLLAPQNTVSSEETD-DFKQETLPSKSNESQDHTDEVD--D-DDE

TR|G3VEM2|G3VEM2_SARHA NFVATWLNADPSQKQTLLATQNSLSSEEST-EDLQETLPRNSSESPDDIDDEDDD---DG

TR|F7DN59|F7DN59_CALJA DAVATWLKPDPSQKQNLLAPQNAVSSEETD-DFKQETLPSKSNESHDDMDDVD--DDDDD

TR|I3MYE1|I3MYE1_SPETR DAVATWLKPDPSQKQNLLAPQNAVSSEERD-DFKQETLPSKSNESHDHMDDVD--DDDDG

TR|F7EC46|F7EC46_ORNAN NQLSSWLNTDPSQKQALLAPQNLVSSEESKENIQQQTLPSISNESHDDVDDADDQ-----

TR|F7ANS8|F7ANS8_MONDO NLVATWLNADPSQKQTLLATQNSVSSEEST-EDLQQTLPSNSNESPDTTDDVDDD--DDD

SP|P10451|OSTP_HUMAN DHVDSQDSIDSNDSDDVDDTDDSHQSDESHHSDESDELVTDF----PTDLPATEVFTPVV 150

SP|P08721|OSTP_RAT DHAESEDSVNSDE------------SDESHHSDESDESFTAS--------TQADVLTPIA

SP|P10923|OSTP_MOUSE DHAESEDSVDSDE------------SDESHHSDESDETVTAS--------TQADTFTPIV

SP|P31096|OSTP_BOVIN S-----QDVNSNDSDDAETTDDPDHSDESHHSDESDEVD--F----PTDIPTIAVFTPFI

SP|P14287|OSTP_PIG DHVDSRDT----DSEEADHADDADRSDESHHSDESDELVTDF----PTDTPATD-VTPAV

TR|F6V2X3|F6V2X3_MACMU DHVDSQDSIDSNDSDEVDDTDDSHQSDESHHSDESDELVTDF----PTDLPATEVFTPVV

TR|H2RCV1|H2RCV1_PANTR DHVDSQDSIDSNDSDDVDDTDDSHQSDESHHSDESDELVTDF----PTDLPATEVFTPVV

TR|G3QS39|G3QS39_GORGO DHVDSQDSIDSNDSDDVDDTDDSHQSDESHHSDESDELVTDF----PTDLPATEVFTPVV

SP|P31097|OSTP_RABIT DHVDNRDSN---ESDDADHPDDSHHSDESHQSDESDEVTV-Y----PTEDAATTVFTEVV

SP|Q9XSY9|OSTP_SHEEP S-----QEVNSDDSDDAETPDDSDHSNESHHSDESDEAD--F----PTDIPTIAVFTPPF

TR|F7AYC1|F7AYC1_HORSE DHEDDQDSIDSDDSDETDPTDDPDNSDESHHSDESDELVTDF----STDVPATPVFTPAV

TR|M3VZ83|M3VZ83_FELCA DDVDSQDSVDSHDTDD-----------DSNQSDESDELVTDF----PTDVPATQFFTPAV

TR|E2R161|E2R161_CANFA DDVDSQDSVDSNDLDD-----------DSNESDESDELVTDF----PTDIPATQLFTPAV

TR|G3SYB7|G3SYB7_LOXAF DHVDSQDSVDSDDLDDDDHTDDPYHSDESHHSDESDELVTGF----PTDDPGTPVFTPAV

TR|Q0WX06|Q0WX06_MESAU DHANSQDSVDSNESDDDDHPDD-DHPDDSHHSDESDESVTAT--------TQTEVFTPAV

TR|U5Y6U2|U5Y6U2_CAPHI R-----HEVNSNDSDDAKTPDDSDHSNESHHSDESDEAD--F----PTDIPTIAVFTPPF

TR|G1PFK5|G1PFK5_MYOLU DRVDSQDSIDSNDSDDDDRTDDPDNSDESHHSDESDELVTDF----PTDFPGTPFATPAV

TR|L5M309|L5M309_MYODS DHVDSQDSIDSNDSDDDDHTDDPDNSDESHHSDESDELVTDFPTDFPTDFPGTPFATPAV

TR|L5KPG6|L5KPG6_PTEAL DHVDSQDHIDSDDSDDADHTDDSDNSDESHHSDESDELVTDF----PTDFPGTPVVTPAV

TR|S7MMZ5|S7MMZ5_MYOBR DHVDSQDSIDSNDSDDDGHTDDPDNSDESHHSDESDELVTDF----PTDFPGTPFATPAV

TR|G3VEM2|G3VEM2_SARHA DHNKS---------------------IDSDDSDESDEVVTDL----PTDTPATPSFLPDG

TR|F7DN59|F7DN59_CALJA DHVDSQD---SNDSDEVDHTDDSHQSDESHHSDESDELVTDF----PTDLPATEVFTPPV

TR|I3MYE1|I3MYE1_SPETR DHVNNQDSTDSDDSDE----DDSHHSDESHHSDESDEVVTES----PTDGPDTPVFTQIV

TR|F7EC46|F7EC46_ORNAN --------------------DDSDHKDESDDSDESDEVVTDF----PTDVPATAVFTPAA

TR|F7ANS8|F7ANS8_MONDO DGHKS---------------------TDSDDSDESDEVVTDF----PTDIPTTSSFLPDG

154/156/157

164

SP|P10451|OSTP_HUMAN PTVDTYDGRGDSVVYGLR-SKSKKFRRPDIQYPDATDEDITSHMESEELNGAY---KAIP 190

SP|P08721|OSTP_RAT PTVDVPDGRGDSLAYGLR-SKSRSFPVSDEQYPDATDEDLTSRMKSQESDEAI---KVIP

SP|P10923|OSTP_MOUSE PTVDVPNGRGDSLAYGLR-SKSRSFQVSDEQYPDATDEDLTSHMKSGESKESL---DVIP

SP|P31096|OSTP_BOVIN PTESANDGRGDSVAYGLK-SRSKKFRRSNVQSPDATEEDFTSHIESEEMHDAP-------

SP|P14287|OSTP_PIG PTGDPNDGRGDSVVYGLR-SKSKKFRRSEAQQLDATEEDLTSHVESEETDGTP---KAIL

TR|F6V2X3|F6V2X3_MACMU PTVDIYDGRGDSVAYGLR-SKSKKFRRPDIQYPDATDEDITSHVESEELNGAY---KAIP

TR|H2RCV1|H2RCV1_PANTR PTVDTYDGRGDSVVYGLR-SKSKKFRRPDIQYPDAIDEDITSHMESEELNGAY---KAIP

TR|G3QS39|G3QS39_GORGO PTVDTYDGRGDSVVYGLR-SKSKKFRRPDIQYPDATDEDITSHMESEELNGAY---KAIP

SP|P31097|OSTP_RABIT PTVETYDGRGDSVAYRLKRSKSKMFHVSNAQYPGASEEDLSSHVDSEDLDDTP---RAIP

SP|Q9XSY9|OSTP_SHEEP PTESTNDGRGDSVAYGLK-SKSKKFRRSNVESPDATEEDFTSHIESEEMHDAP-------

TR|F7AYC1|F7AYC1_HORSE PTRDTYDGRGDSLSYGLK-SKSRKFRRSEDQYPDATEEDLTSPVESKDIDDVH---KAVL

TR|M3VZ83|M3VZ83_FELCA PTRDSYDGRGDSVAYGLR-SKSKKSHRYEDQYPDSTEEDFTSLVKSQSMEDDF---NAVL

TR|E2R161|E2R161_CANFA PTRGSYDGRGDSVAYGLR-SKSKKSHKYEVQYPDSTEEDFTSLVKSASMEDDF---NAVL

TR|G3SYB7|G3SYB7_LOXAF PTVDTYDGRGDNVVYRLS-SKSKKSHKSVIQFPDATEEDFTSRMESKELDDAH---KAIR

TR|Q0WX06|Q0WX06_MESAU PTVEIPDGRGDSLAYGLR-AKSRKFHISDDQYPDTTDEDLSSHMKSKELVDTL---KVIP

TR|U5Y6U2|U5Y6U2_CAPHI PTESTNDGRGDSVAYGLK-SKSKKFRRSNVESPDATEEDFTSHIESEEMHDAP-------

TR|G1PFK5|G1PFK5_MYOLU PTIDTNDGRGDSVAYGLR-LKSKKLHRSVVQYPDATEEDLTSHMESDEMDDAH---RAIL

TR|L5M309|L5M309_MYODS PTIDTNDGRGDSVAHGLR-LKSKKLHRSVVQYPDATEEDLTSHMESDEMDDAH---RAVL

TR|L5KPG6|L5KPG6_PTEAL PTGDTNDGRGDSVAYRLK-SKSKKFHRSVVKYPDATEEDLTSHVESKEVDDARKAVKAVF

TR|S7MMZ5|S7MMZ5_MYOBR PTIDTNDGRGDSVAYGLR-LKSKKLHRSVVQYPDATEEDLTSHMESDEMDDAH---RAIL

TR|G3VEM2|G3VEM2_SARHA PTRGDNGGRGDSVAYGLR-SKLGAPYRSSEQVHDVTEEDLTSQIESYESEKTH---KAIP

TR|F7DN59|F7DN59_CALJA PTIDTYDGRGDSVGYGLR-SKSKKFRRSDIQYPDATEEDITSHVESEELNGAY---KAIP

TR|I3MYE1|I3MYE1_SPETR PTVETYDGRGDSVDYGVR-SKSKKFYISEVQYPDATDEDLTSHMESSELNDAH---KAIP

TR|F7EC46|F7EC46_ORNAN PTRGDNGGRGDRVYRGLK-TKPGVLYKAAVQGHDAS-DDFTSRLESLESDESP---EAYP

TR|F7ANS8|F7ANS8_MONDO PTRGDNSGRGDSVAYGLR-SKVGAPDISSAETHEVTEEDLTSQMESYESEKAH---KAFP

SP|P10451|OSTP_HUMAN VAQDLNAPSDWDSRGKD--------------SYETSQLDDQSAETHSHKQSRLYKRKAND 236

225/226

231

213

217

193

206

SP|P08721|OSTP_RAT VAQRLSVPSDQDSNGKT--------------SHESSQLDEPSVETHSLEQSKEYKQRASH

SP|P10923|OSTP_MOUSE VAQLLSMPSDQDNNGKG--------------SHESSQLDEPSLETHRLEHSKESQ-----

SP|P31096|OSTP_BOVIN --------------------------------KKTSQLTDHSKETNSSELSKELTPKAKD

SP|P14287|OSTP_PIG VAQRLHVASDLDSQEKD--------------SQETSQPDDRSVETRSQEQSKEYTIKTYD

TR|F6V2X3|F6V2X3_MACMU VVQGLNVPSDWDSRGKD--------------SHETSQLDDHSAETHSHKHSRLYKRKASD

TR|H2RCV1|H2RCV1_PANTR VAQDLNAPSDWDSRGKD--------------SYETSQLDDQSAETHSHKQSRLYKRKASD

TR|G3QS39|G3QS39_GORGO VAQDLNAPSDWDSRGKD--------------SYETSQLDDQSAETHSHKQSRLYKRKASD

SP|P31097|OSTP_RABIT VAQHLNVPSDWDSQEKD--------------SHDVSQVDDHSVETQSHEQARQYKREAND

SP|Q9XSY9|OSTP_SHEEP --------------------------------KKTSQLTDHSEETNSDELPKELTPKAKE

TR|F7AYC1|F7AYC1_HORSE VAQGLHVASDWDSRGKD--------------SQETSQLDDHSVETHSREHSKEYQLKAND

TR|M3VZ83|M3VZ83_FELCA LSHTVRRSPDRDSHVKD--------------SQETSQVDDHSMETKSRKHSKEYKLKASD

TR|E2R161|E2R161_CANFA LSRTVRGTSDRDSHAKD--------------SQETSQLDDHSMETKGRKHSQEYKLRASD

TR|G3SYB7|G3SYB7_LOXAF VVKSLKRASDWDSHQQD--------------SHETSQLDDHSVETHSQEQSKEYKRKAND

TR|Q0WX06|Q0WX06_MESAU VAYRLNEPSDQDSTGKT--------------SHESSQLDEPSVETHSHEQSQEHKQKASH

TR|U5Y6U2|U5Y6U2_CAPHI --------------------------------KKTSQLTDHSEETNSDELSKELTPKAQE

TR|G1PFK5|G1PFK5_MYOLU VAQGLPVASDWDSRGKD--------------SQETSQLDDHSVETHSHEAAKEYKLKAMD

TR|L5M309|L5M309_MYODS VAQGLPVASDWDSRGKD--------------SQETSQLDDHSVETHSHELAKGYKLKAMD

TR|L5KPG6|L5KPG6_PTEAL VAQGLHVASDWDSPRKD--------------SQETSQLDDHSTETHSLEHSEEYKLKAND

TR|S7MMZ5|S7MMZ5_MYOBR VAQGLPVASDWDSQGKD--------------SQEASQLDDHSVETHSREAAKEYKLKAMD

TR|G3VEM2|G3VEM2_SARHA LSQTFPKVSSWESNGQE--------------SNEASQADEYSVETHSHEQLKSSQLERNI

TR|F7DN59|F7DN59_CALJA VAQSLNVPSDWDSHGKD--------------SHEMSQLDDQSAETHSHEQSRLHKRKASD

TR|I3MYE1|I3MYE1_SPETR VAHRLKVPSDWDSHGKD--------------SHETSQLDDHSVETHSQEQTREYKRNARD

TR|F7EC46|F7EC46_ORNAN DAHKLQKSSEWHSNEASHQDDRSMQKSSEWHSSEASHQDDRSVETHSHEEAKGYRLKQED

TR|F7ANS8|F7ANS8_MONDO LSQNLPKVSAWGSNSKE--------------SNEASHPDEYSVETYSHEQFKSYQLEETN

248

252

SP|P10451|OSTP_HUMAN ESNEHS------------------DVIDSQELSKVSREFHS------------------- 259

SP|P08721|OSTP_RAT ESTEQSDAIDSAEKPDAIDSAERSDAIDSQASSKASLEHQS-------------------

SP|P10923|OSTP_MOUSE ------------------ESADQSDVIDSQASSKASLEHQS-------------------

SP|P31096|OSTP_BOVIN -KNKHS------------------NLIESQENSKLSQEFHS-------------------

SP|P14287|OSTP_PIG GSNEHS------------------NVIESQENPKVSQEFHS-------------------

TR|F6V2X3|F6V2X3_MACMU DSNEHS------------------DVIDSQERSKISHEFHS-------------------

TR|H2RCV1|H2RCV1_PANTR ESNEHS------------------DVIDSKELSKVSREFHS-------------------

TR|G3QS39|G3QS39_GORGO ESNEHS------------------DVIDSQELSKVSREFHS-------------------

SP|P31097|OSTP_RABIT NSVEHS------------------HSIDSQESSKVSQESQS-------------------

SP|Q9XSY9|OSTP_SHEEP -ESKHS------------------NRIESQENSKLSQEFHS-------------------

TR|F7AYC1|F7AYC1_HORSE ETSEHS------------------DVIDSRENSKVSQEFPS-------------------

TR|M3VZ83|M3VZ83_FELCA ENNKHS------------------HEIGSQESSDISSELVG-------------------

TR|E2R161|E2R161_CANFA ESNMHS------------------HEIGSQENSEVSSELVS-------------------

TR|G3SYB7|G3SYB7_LOXAF VSSEHS------------------DMIDSQENSKVSHEFHS-------------------

TR|Q0WX06|Q0WX06_MESAU ESTELSD------------------VIDSQESSKASQEHQ--------------------

TR|U5Y6U2|U5Y6U2_CAPHI -ESKHS------------------NRIESQENSKLSQEFHS-------------------

TR|G1PFK5|G1PFK5_MYOLU ASNEHS------------------VGIDSQENSRVSHEFHS-------------------

TR|L5M309|L5M309_MYODS ASNEHS------------------VGIDSQENSRVSQEFHS-------------------

TR|L5KPG6|L5KPG6_PTEAL ESNGHA------------------DVIDSQENSKVSHEFHS-------------------

TR|S7MMZ5|S7MMZ5_MYOBR ASNEHS------------------VGIDSQENSRVSHEFHS-------------------

TR|G3VEM2|G3VEM2_SARHA YDSQ-----------------QQSDSHGSQENDKVSQEFHNREVDRDSQEFHKQQVGKLS

TR|F7DN59|F7DN59_CALJA ESNEHS------------------DVIDSQEVSKVSREFHS-------------------

TR|I3MYE1|I3MYE1_SPETR NSSEHS------------------DVIDSQESSRVSQEFQS-------------------

TR|F7EC46|F7EC46_ORNAN HSSQQD------------------DLNDSQESYKVSRENDSQEKT---------------

TR|F7ANS8|F7ANS8_MONDO PDSQ-----------------QQGDSHDSQENDKVSQEFRT------------QEVNKLS

283

SP|P10451|OSTP_HUMAN HEFHSHEDMLVVDPKSKEEDKHLKFRISHELDSASSEVN 298

SP|P08721|OSTP_RAT HEFHSHEDKLVLDPKSKEDDRYLKFRISHELESSSSEVN

SP|P10923|OSTP_MOUSE HKFHSHKDKLVLDPKSKEDDRYLKFRISHELESSSSEVN

SP|P31096|OSTP_BOVIN -----LEDKLDLDHKSE-EDKHLKIRISHELDSASSEVN

SP|P14287|OSTP_PIG -----HEDKLVPDSKSE-EDKHLKLRVSHELESASSEIN

TR|F6V2X3|F6V2X3_MACMU HEFHSHEDMLVVDPKSKEEDKHLKFRISHELDSASSEVN

TR|H2RCV1|H2RCV1_PANTR HEFHSQGDMLVVDPKSKEEDKHLKFRISHELDSASSEVN

TR|G3QS39|G3QS39_GORGO HEFHSHEDMLVVDPKSKEEDKHLKFRISHELDSASSEVN

SP|P31097|OSTP_RABIT REFRSHEDKLAIEPKSEEDEEHRQLRVSHELDSTSSEIN

SP|Q9XSY9|OSTP_SHEEP -----LEDKLDLDHKSE-EDKRLKIRISHELDSVSSEVN

TR|F7AYC1|F7AYC1_HORSE QEFHSSEGKLVRDRKSEDEDKYLKFRTSHESESASSEVN

TR|M3VZ83|M3VZ83_FELCA QTVQSNEKELVQHPESEEQDKHLKFRVSHELDSASSEVN

TR|E2R161|E2R161_CANFA QLSQSHEKELIVDSKSEEEDKHLKFHVSHELDSASSEIN

TR|G3SYB7|G3SYB7_LOXAF -----HEDKHRPDPESEEQDKHLKFLISHELESKLEKKN

TR|Q0WX06|Q0WX06_MESAU ----SHEDKLVPDFKSKEDTNHLKIRISHEIESSSSEVN

TR|U5Y6U2|U5Y6U2_CAPHI -----LEDKLDLDHKSE-EDKRLKIRISHELDSASSEVN

TR|G1PFK5|G1PFK5_MYOLU RELQSHEDKLASAPQSEEQDKHLKFRISHELESASSEVN

TR|L5M309|L5M309_MYODS RELQSHEDKLASDPQSEEQDKHLKFRISHELESASSEVN

TR|L5KPG6|L5KPG6_PTEAL QEFDSREDKLALDPKSEDQDKHLKFRISHELESASSEVN

TR|S7MMZ5|S7MMZ5_MYOBR RELQSHEDKLASAPQSEEQDKHLKFRISHELESASSEVN

TR|G3VEM2|G3VEM2_SARHA QEYHSQEVHLVSDPESVENIKPLKLHSHE-VDSASFETH

TR|F7DN59|F7DN59_CALJA HEFHSQEDLLVVDPKSTEEDKHLKFRISHELDSASSEVN

TR|I3MYE1|I3MYE1_SPETR HESHSKEDKLVLDPKSK-EDKHLIFHVSHEIESASSEVN

TR|F7EC46|F7EC46_ORNAN --------AQDADSDEFNRKHYLKSHTSHEFDSASSETH

TR|F7ANS8|F7ANS8_MONDO QEFQSQESHPVNDPESVENTKYLKLHSFPEADSASYEAH
