# Supplementary material for: Identification of Transglutaminase Reactive Residues in Human Osteopontin and Their Role in Polymerization
Source: PLoS One. 2014 Nov 24;9(11):e113650. doi: 10.1371/journal.pone.0113650 (PMC4242673; doi:10.1371/journal.pone.0113650)
Supplement: Table S1 — Identification of reactive Gln residues in human milk OPN and recombinant OPN. OPN was labeled with 5-(Biotinamido)pentylamine for 15 min or 5 h by TG2. Identified reactive Gln residues are underlined and shown in bold. The ppm differences between the measured and expected masses are listed. (DOCX) [file pone.0113650.s002.docx]

**Table S1.** Identification of reactive Gln residues in native human milk OPN and recombinant OPN. OPN was labeled with 5-(Biotinamido)pentylamine for 15 min or 5 h by TG2. Identified reactive Gln residues are underlined and shown in bold. The ppm differences between the measured and expected masses are listed.

|  | Reactive Gln | Peptide | ppm |
| --- | --- | --- | --- |
| Milk OPN/15 min | 34 | YPDAVATWLNPDPS**Q**K | 5.9 |
|  | 42 | QNLLAP**Q**NAVSSEETNDFK | 5.0 |
|  | 193 | AIPVA**Q**DLNAPSDWDSR | 3.7 |
| Milk OPN/5 h | 5 | **Q**ADSGSSEEK | 3.3 |
|  | 34 | YPDAVATWLNPDPS**Q**K | 6.7 |
|  | 36 | **Q**NLLAPQNAVSSEETNDFK | 7.1 |
|  | 42 | QNLLAP**Q**NAVSSEETNDFK | 5.6 |
|  | 55 | **Q**ETLPSK | 2.8 |
|  | 193 | AIPVA**Q**DLNAPSDWDSR | 6.3 |
|  | 213 | GKDSYETS**Q**LDDQSAETHSHK | 0.1 |
|  | 217 | GKDSYETSQLDD**Q**SAETHSHK | 0.7 |
|  | 248 | KANDESNEHSDVIDS**Q**ELSK | 1.9 |
| Recombinant OPN/5 h | 5 | **Q**ADSGSSEEK | 4.5 |
|  | 34 | YPDAVATWLNPDPS**Q**K | 6.9 |
|  | 36 | **Q**NLLAPQNAVSSEETNDFK | 6.4 |
|  | 42 | QNLLAP**Q**NAVSSEETNDFK | 6.4 |
|  | 55 | **Q**ETLPSK | 2.0 |
|  | 164 | RPDI**Q**YPDATDEDITSHMESEELNGAYK | 5.4 |
|  | 193 | AIPVA**Q**DLNAPSDWDSR | 4.6 |
|  | 213 | GKDSYETS**Q**LDDQSAETHSHK | 3.5 |
|  | 217 | GKDSYETSQLDD**Q**SAETHSHK | 3.6 |
|  | 248 | KANDESNEHSDVIDS**Q**ELSK | 4.6 |
